# Impact of different cephalometric skeletal configurations on anatomic midface parameters in adults

**DOI:** 10.1007/s00784-023-05472-7

**Published:** 2023-12-29

**Authors:** Ines Willershausen, Amelie Ehrenfried, Franziska Krautkremer, Armin Ströbel, Corinna Lesley Seidel, Friedrich Paulsen, Markus Kopp, Michael Uder, Lina Gölz, Matthias Stefan May

**Affiliations:** 1 Department of Orthodontics and Orofacial Orthopedics, Friedrich-Alexander-University Erlangen-Nürnberg, Gluecksstrasse 11, 91054 Erlangen, Germany; 2 Center for Clinical Studies (CCS), Medical Faculty, Friedrich-Alexander University Erlangen-Nuremberg, University Hospital Erlangen, Erlangen, Germany; 3 Institute of Functional and Clinical Anatomy, Friedrich-Alexander-University Erlangen-Nürnberg, Erlangen, Germany; 4 Institute of Radiology, Friedrich-Alexander-University Erlangen-Nürnberg, Erlangen, Germany

**Keywords:** Midface, Anatomy, Computed tomography, Cephalometry, Orthodontics, Ultra-high-resolution datasets

## Abstract

**Objectives:**

Skull morphology and growth patterns are essential for orthodontic treatment, impacting clinical decision making. We aimed to determine the association of different cephalometric skeletal configurations on midface parameters as measured in 3D CT datasets.

**Materials and methods:**

After sample size calculation, a total of 240 fully dentulous patients between 20 and 79 years of age (mean age: 42 ± 15), who had received a CT of the skull within the scope of trauma diagnosis or intracranial bleeding, were retrospectively selected. On the basis of cephalometric analysis, using MPR reconstructions, patients were subdivided into three different vertical skull configurations (brachyfacial, mesofacial, dolichofacial) and the respective skeletal Class I, II, and III relationships. Anatomic parameters were measured using a three-dimensional post-processing console: the thickness of the maxillary and palatine bones as well as the alveolar crest, maxillary body and sutural length, width and height of the hard palate, maxillary facial wall thickness, and masseter muscle thickness and length.

**Results:**

Individuals with brachyfacial configurations had a significantly increased palatal and alveolar ridge thicknesses compared to those with dolichofacial- or mesofacial configurations. Brachyfacial configurations presented a significantly increased length and thickness of the masseter muscle (4.599 cm; 1.526 cm) than mesofacial (4.431 cm; 1.466 cm) and dolichofacial configurations (4.405 cm; 1.397 cm) (*p* < 0.001). Individuals with a skeletal Class III had a significantly shorter palatal length (5.313 cm) than those with Class I (5.406 cm) and Class II (5.404 cm) (*p* < 0.01). Sutural length was also significantly shorter in Class III (*p* < 0.05).

**Conclusions:**

Skeletal configurations have an impact on parameters of the bony skull. Also, measurable adaptations of the muscular phenotype could result.

**Clinical relevance:**

The association between viscerocranial morphology and midface anatomy might be beneficial for tailoring orthodontic appliances to individual anatomy and planning cortically anchored orthodontic appliances.

## Introduction

The maxillary complex, which comprises the premaxillary as well as the maxillary and palatine bones, is a landmark of the midface with utmost importance for orthodontic treatment planning [1]. A profound knowledge of midface anatomy is indispensable for any treatment devoted to the post-development of the upper jaw in the transversal, sagittal, and vertical plane [2–4]. A transversal expansion of the upper jaw is necessary for severe maxillary constrictions or crossbites, with chronological age and skeletal features contributing to an increased likelihood of transversal resistance [5, 6]. Among others, mid-palatal suture maturation is responsible for the lack of success of transversal expansion, with an increased probability of unfavourable dental side effects, such as buccal tipping, recessions, and gingival ulcers [7–9]. The abovementioned factors determine, whether a primarily tooth-borne rapid maxillary expansion device (RME) should be additionally or solely anchored cortically. A micro-implant-assisted rapid palatal expansion (MARPE) utilizes both, the hard palate and the dentition as an anchorage, while a bone-borne distractor directly transfers the orthodontic forces to the palatal bone [10, 11].

Apart from transversal expansion, multiple orthodontics conditions call for maximum skeletal anchorage, with the hard palate and the interradicular space of the alveolar crest being preferred insertion sites for orthodontic implants [12, 13]. Orthodontic implants have experienced an increasing popularity over the last decades, improving anchorage and expanding treatment options for adult patients [14, 15]. Consequently, knowledge of the respective anatomy is crucial for the accurate positioning and stability of orthodontic implants, avoiding premature implant loss. Due to its great importance for skeletal anchorage, palatal thickness has been investigated intensively in literature [16–20]. Concerning the hard palate, the T-zone, which describes the area immediately posterior to the palatal rugae, has been validated as a reliable location with sufficient bone thickness [21]. Overall, palatal thickness has been reported to be most extensive in the anterior part of the palatal vault, comprising median and paramedian areas of the anterior hard palate, as opposed to posterior areas of the hard palate [12]. Nevertheless, a tremendous inter-individual inhomogeneity for palatal thickness has been depicted in the literature [20, 22]. Explanations alluded to in the literature are that palatal thickness is most likely influenced by a plethora of co-factors such as maxillary body length, sex, and age [17, 18, 20].

It has been described that bone thickness of the alveolar crest is equally influenced by age, sex, and craniofacial growth patterns [23]. Notably, vertical facial growth patterns have a more significant impact than the respective sagittal relation, with the highest alveolar bone thickness found in hypodivergent individuals [24, 25].

Besides bony features of the midface, soft-tissue landmarks such as the muscle thickness of the masseter are determined by the respective viscerocranial configurations, with the thickest muscles found in brachyfacial phenotypes [26]. The present study investigates the influence of vertical skull configurations (brachyfacial, mesofacial, dolichofacial) as well as the respective skeletal Class I, II, and III relationships on predefined hard and soft tissue parameters of the midface.

## Materials and methods

We retrospectively investigated 240 patients, who had received a diagnostic CT of the skull concerning soft and hard tissue parameters of the midface. Before enrolling in this study, the institutional review board had given their positive consent (IRB Number: 22–174-Br). In a period from May 2021 to May 2022, CT datasets from 240 patients of European descent (m = 110 (54%), f = 130 (46%)), 20–79 years of age (mean age: 42 ± 15), who were examined within the scope of cranial trauma, inflammatory disease or tumor staging, were retrospectively selected from our archives. We enclosed CT datasets with high resolutions down to 0.6 mm slice thickness and a 512 pixel matrix (SOMATOM X.cite, Siemens Healthcare GmbH, Forchheim, Germany), and ultra-high resolution using a dedicated filter at the detector side to obtain 0.4 mm slice thickness combined with a maximum of 1024 pixel matrix (SOMATOM X.ceed, Siemens Healthcare GmbH, Forchheim, Germany). Ultra-high-resolution datasets were chosen preferably. Patients were only enclosed if they had a maximum of one singular tooth missing per jaw. Subjects with facial skull asymmetries, neoplasms of the skull, craniofacial malformations such as orofacial clefts, displaced teeth in the palatal region, and metabolic disorders of the bones were also excluded.

**Fig. 1:**
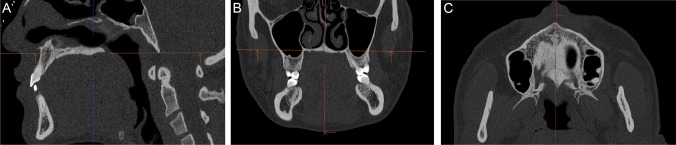
Standardized alignment of the CT data set in **A**) the sagittal, **B**) the coronal, and **C**) the axial slices according to previously defined reference lines and reference points. The following parameters were measured in the respective slices: **A**) sagittal slice: palatal length; **B**) coronal slice: median and paramedian palatal thickness, alveolar crest thickness; **C**) axial slice: mid-palatal suture length, maxillary sinus facial wall thickness, width of pterygomaxillary junction, length and thickness of the masseter muscle

All CT data sets were evaluated with a three-dimensional post-processing console (Syngo.via VB60_A, Siemens Healthcare GmbH, Erlangen, Germany). This software offers the possibility to align the datasets in a standardized way in all three spatial planes ensuring a standardized evaluation. Each CT was aligned in the axial, coronal, and sagittal slices according to predefined reference points and planes to ensure reproducibility. The midline of the face served as a reference in the coronal slice, and the spinal plane was utilized as a reference line for the axial and sagittal slices (Fig. 1).

If the axis alignment is adjusted in one of the planes, the alignment of the other two axes is also changed since they are always perpendicular to each other.

**Fig. 2:**
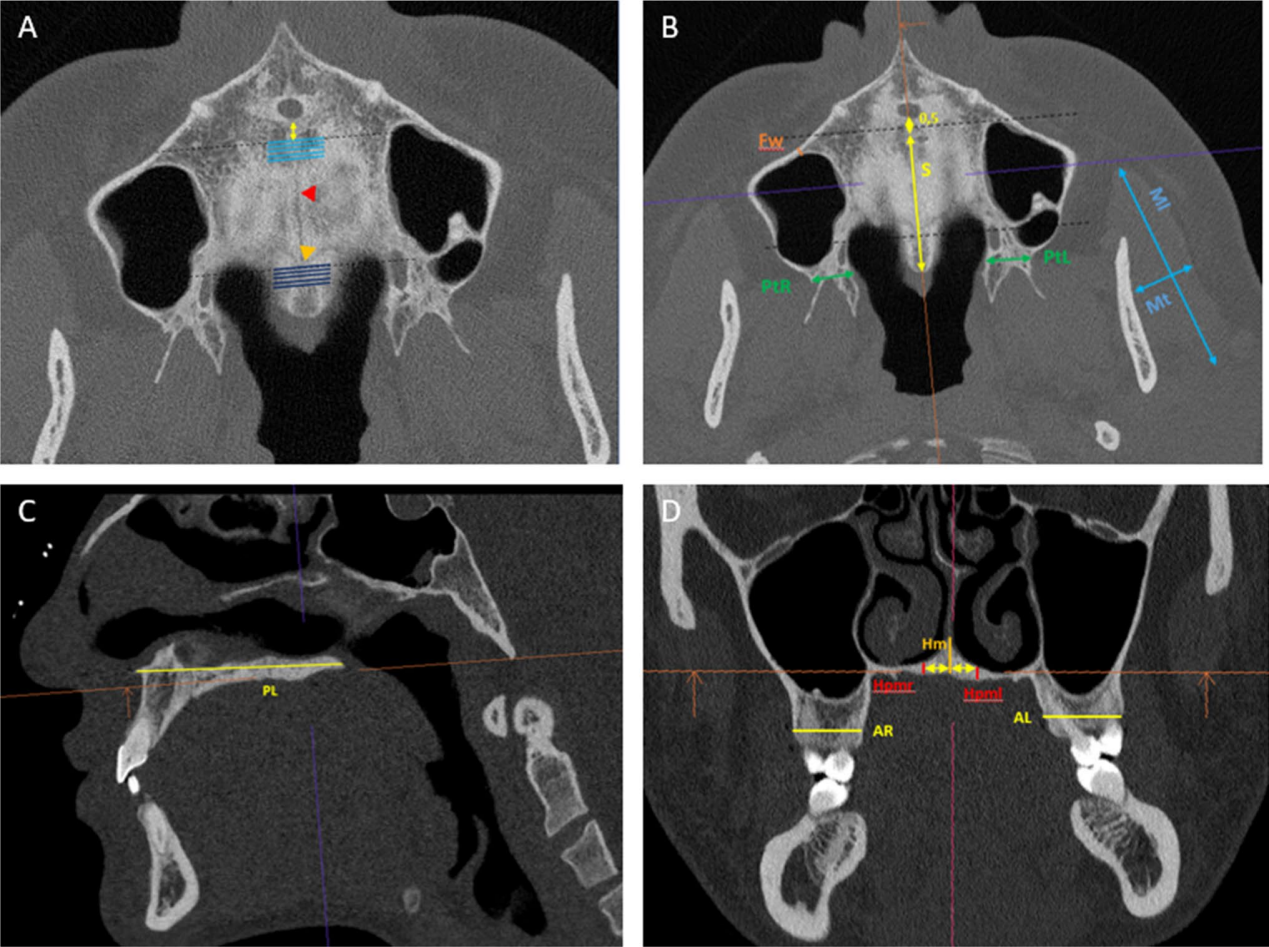
Measurements performed in the axial (**A**, **B**), sagittal (**C**), and coronal slices (**D**) of the CT datasets. **A**: Anterior (light blue lines) and posterior palatal thickness (dark blue lines) were measured in 5 consecutive slices in the maxillary and palatine bone. The mid-palatal suture (red arrowhead), as well as the transverse palatine suture (orange arrowhead), are depicted. **B**: Further measurements conducted within the axial slices are the thickness of the maxillary facial wall (Fw), the length of the mid-palatal suture (S), the thickness of the pterygomaxillary junction (PtL/R), as well as the length and thickness of the masseter muscle (Ml, Mt). **C**: Palatal length (PL) was determined in the sagittal slice. **D**: Median (Hm) and paramedian (Hpmr/l) palatal thickness, as well as the thickness of the alveolar crest, were measured within the coronal slices

A total of 106 variables were measured on every single CT dataset, resulting in 25.440 measurements for the entire investigation collective of 240 patients without any missing values. The measurements, which were performed within the axial slice of the three-dimensional dataset, comprised the length of the hard palate (Fig. 2B, C), ranging from the anterior to the posterior spine of the hard palate, the length of the mid-palatal suture starting at 0.5 mm dorsal to the incisive foramen, the thickness of the maxillary sinus facial wall and the width of pterygomaxillary junction (Fig. 2B).

In the coronal slice, the palatal thickness of both, the maxillary and the palatine bones, was measured in the midline of the hard palate (mid-palatal sutural region) and 0.25 cm lateral of the suture on the right and left side of the hard palate (Fig. 2D). The palatal thickness of the maxillary was measured starting 0.5 mm dorsal of the incisive foramen, with five subsequent measurements being performed in the consecutive coronal CT slices. The measurements of the palatal thickness in the palatine bones were performed accordingly, being initiated 0.5 mm dorsal to the transverse palatine suture. All measurements of palatal thickness were strictly conducted at a 90° angle to the spinal plane (Fig. 2A, D). Furthermore, the thickness of the alveolar ridge in the molar and premolar region, both on the left and right side of the face, was measured in the coronal view (Fig. 2D). With regard to soft tissue parameters, the length and thickness of the masseter muscle were measured on the left and right sides of the face (Fig. 2B).

A cephalometric analysis was carried out using a sagittal thick slice MPR reconstruction as an alternative for a lateral radiograph, which was imported into Onxy Ceph (Image Instruments, Germany), where a cephalometric analysis was carried out [27, 28]. Based on the outcome of the lateral cephalometry, employing the cephalometric angles NL-NSL, ML-NL, ML-NSL, Me-tgo-Ar and the Jarabak index, every patient was assigned to either a brachyfacial, mesofacial, and dolichofacial skull configuration [28, 29]. Furthermore, based on SNA, SNB, ANB, and Wits-values every patient was allocated to skeletal Class I (neutral), Class II (distal) or Class III (mesial) [28–30]. The cephalometric analysis of all 240 MRP reconstructions was performed by three raters with more than three years of expertise in maxillofacial radiology. Based on a preceding statistical power analysis, 50 selected CT datasets were analysed by the abovementioned three raters, calibrated beforehand, to determine the interrater reliability, ensuring the validity of the measurements. Based on the preceding interrater reliability, the remaining 190 CT datasets were evaluated by one rater.

### Statistical analysis

A preliminary data set of 66 patients (29 brachyfacial, 22 mesofacial,15 dolichofacial) was used for the sample size calculation. A total of 59 interval-scaled variables were measured for every patient. For every variable, the effect sizes for the pairwise comparison were estimated, resulting in a total of 177 (= 59 variables*3 groups) effect sizes. The corresponding sample sizes to these effect sizes were calculated by a two-sample two-sided *t*-test with 80% power and a significance level of 5%. These sample sizes ranged from *n* = 20 patients for variables with great effect sizes to *n* >  > 1000 patients for variables with small effect sizes. Twenty-five variables showed effect sizes corresponding to n ≤ 80 samples per group. It was consequently decided to collect a data set with 80 brachyfacial, 80 mesofacial and 80 dolichofacial individuals, resulting in a total of 240 patients for further exploratory data analysis.

Statistical analyses were conducted using R version 4.2.1. For descriptive analyses, means and standard deviations (SD) were calculated for continuous variables and counts and percentages for discrete variables. Overall, 106 variables from 240 patients were measured. Some of these variables were measured only once in every patient (e.g., palatal and sutural length), on both the left and right of the jaw (e.g., maxillary facial wall thickness, masseter muscle thickness and length, pterygomandibular junction, the thickness of the alveolar crest) and in five layers (e.g., the paramedian and median thickness of the maxillary and palatine bones).

These repeated measurements were tested for pooling: the criteria for pooling was a non-significant ANOVA and a high correlation (> 0.75) of the repeated measurements. All variables (pooled and also non-pooled) were tested for a significant effect on the variable vertical skull configuration (brachyfacial, mesofacial, dolichofacial) by ANOVA and post hoc-test Tukey’s Honest Significant Differences [31]. The inter-rater reliability of three raters of 60 variables was estimated by intraclass-correlations [32]. We choose the coefficient ICC (2,1): two-way ANOVA absolute agreement between judge’s ratings.

## Results

### Demographics

The retrospective study population comprised 240 individuals of European descent ranging from 20–79 years of age, with a mean age of 42 ± 15 years. A total of 113 CT datasets had a high resolution and 127 datasets had an ultra-high resolution. One hundred ten patients were male (46%), and 130 were female (54%). According to their cephalometric skull configuration, patients were further subdivided into three groups: brachyfacial, mesofacial, dolichofacial and well as the respective skeletal Class I (neutral), Class II (distal) and Class III (mesial) relation, serving as subgroups.

The collective comprised a total of 82 patients with a mesofacial (34%), 79 patients with a brachyfacial (33%), and 79 patients with a dolichofacial (33%) vertical skull configuration. When subdividing this collective concerning skeletal Classes, a total of 112 patients had a neutral (47%), 73 patients had a distal (30%), and 55 patients had a mesial (23%) relation (Table 1).

**Table 1:** Demographic details of the investigated collective

	Brachyfacial [*n* = 79]	Mesofacial [*n* = 82]	Dolichofacial [*n* = 79]
Distal	39% (31)	22% (18)	30% (24)
Mesial	22% (17)	30% (25)	16% (13)
Neutral	39% (31)	48% (39)	53% (42)
Sex			
Female	41% (32)	57% (47)	65% (51)
male	59% (47)	43% (35)	35% (28)
Age at CT [years]	40.35 ±14.40 (79)	41.07 ±15.45 (82)	44.24 ±13.19 (79)

### Inter-rater reliability

The intraclass correlation coefficient (ICC) of all 60 variables was calculated simultaneously and pairwise for all three raters. Half of the simultaneous ICCs are above 0.82, and 25% are above 0.9. There are a few downward outliers: maxillary facial wall and pterygomaxillary junction showed minor agreement between raters. Apart from that, a high degree of interrater agreement was observed overall. Furthermore, the coefficients of variation, hence IQR (inter-quartile range) and mean/IQR ratio, were calculated. Variables with a high coefficient of variation were especially the cephalometric values like SNA and SNB but also the width of the alveolar crest and palatal thickness.

### Hard tissue parameters

Regarding the length of the palatal plane (PL), no significant differences were found between the brachyfacial, mesofacial or dolichofacial phenotypes. However, the length of the hard palate was significantly shorter in mesial patients than in those with a distal or neutral configuration (*p* < 0.01). The palate was 5.313 cm long on average in mesial patients, while these values increased to 5.404 cm in distal and 5.406 cm in neutral, respectively.

**Fig. 3:**
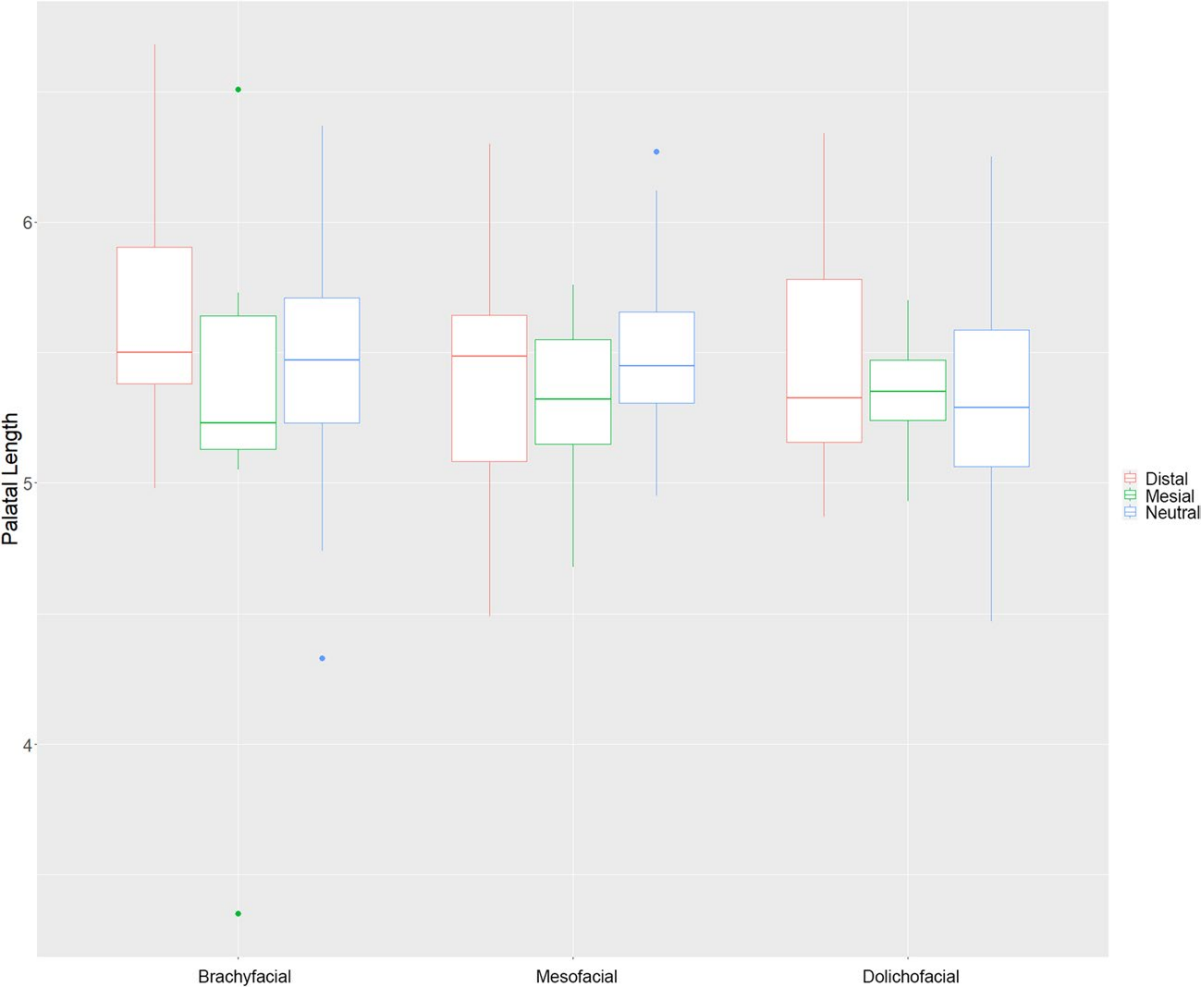
Palatal length in the different subgroups. The red-colored boxplot indicates distal, green mesial, and blue neutral skull configuration

Furthermore, statistically significant differences could be observed between male and female patients (*p* < 0.05). Male patients’ palatal length was 5.632 cm, while those values declined to 5.230 cm in females (Table 2, Fig. 3).

**Fig. 4:**
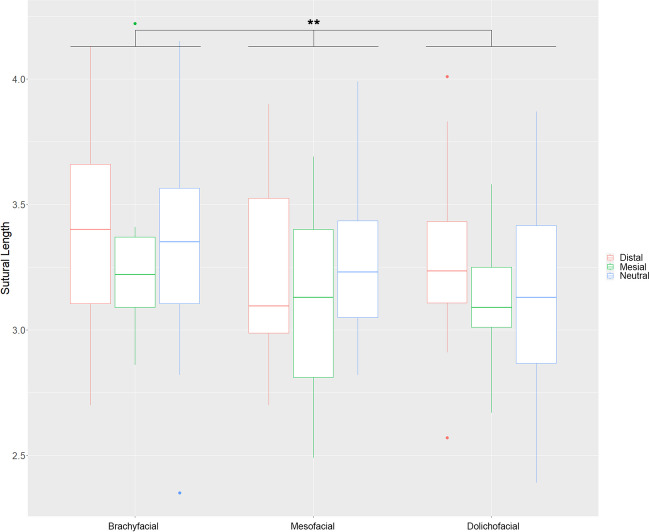
Sutural length in the different subgroups. The red-colored boxplot indicates distal, green mesial, and blue neutral skull configuration. **p < 0.01

As for the length of the mid-palatal suture (S), significant differences are found between vertical facial phenotypes (*p* < 0.01). With regard to pairwise comparisons, patients with a brachyfacial skull configuration (3.348 cm) show a significantly increased a mean sutural length than individuals with a mesofacial (3.203 cm) (*p* = 0.018) and patients with a dolichofacial skull configuration (3.175 cm) (*p* = 0.004). The respective skeletal class relation also appears to impact sutural length; however, the statistical influence is smaller (*p* < 0.05) than in the vertical groups. Patients with a mesial relation have the shortest sutures with 3.153 cm, followed by distal with 3.312 cm, and neutral configurations with 3.394 cm, respectively. Sutural length statistically differed between male and female patients (*p* < 0.001). The male sutural length was 3.368 cm on average, while those values declined to 3.134 cm in females (Table 2, Fig. 4).

**Fig. 5:**
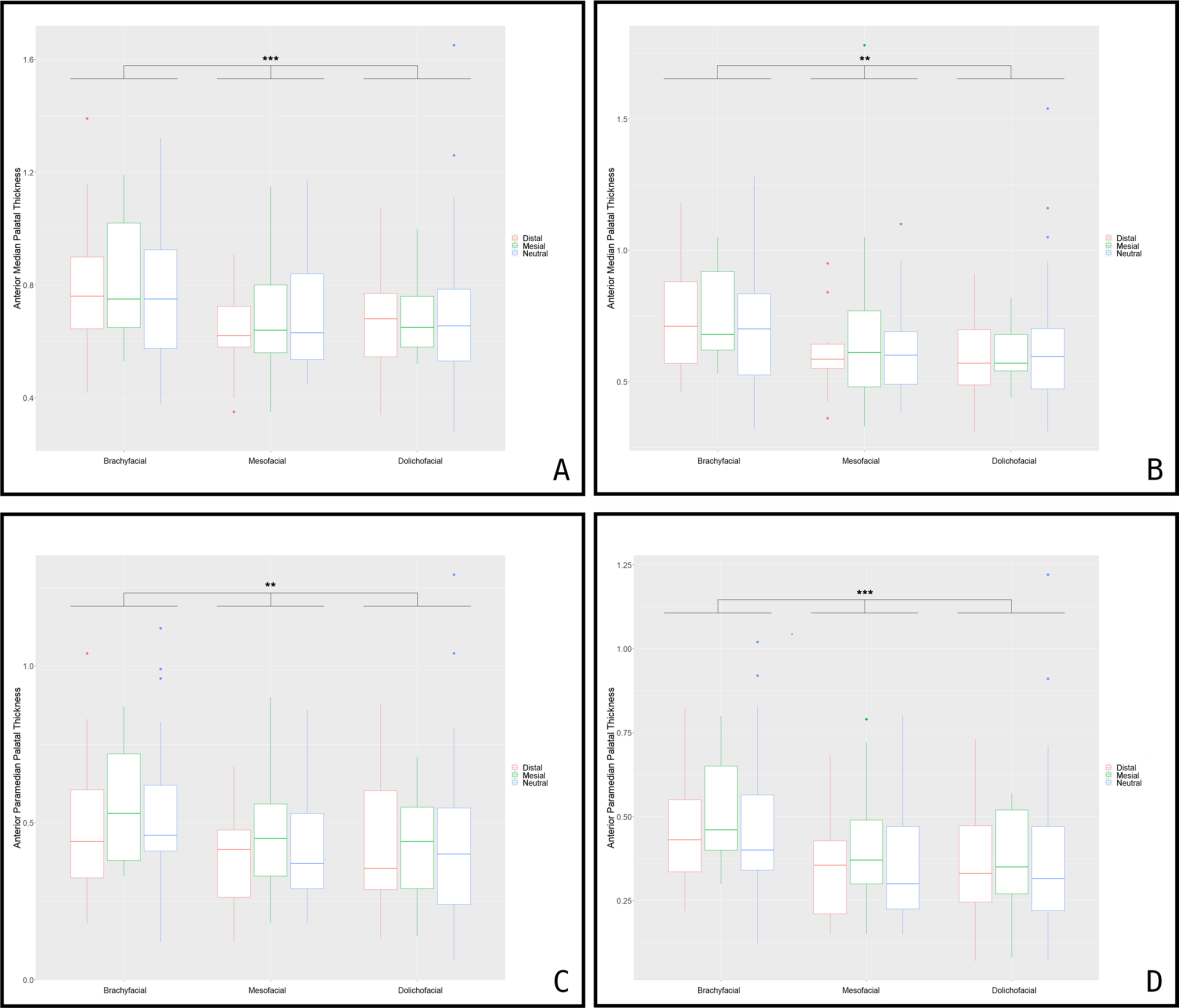
Anterior median palatal thickness as measured in the first slice (**A**) and last slice (**B**). Anterior paramedian palatal thickness is measured in the first slice (**C**) and the last slice (**D**). The red-colored boxplot indicates distal, green mesial, and blue neutral skull configuration. **p < 0.01; ***p < 0.001

The palatal thickness of the maxillary bone significantly decreases from ventral to dorsal throughout the entire collective. This finding alludes to both the median (Hmv (slice1-5)), the left paramedian (Hpmlv (slice1-5)) as well as the right paramedian (Hpmrv (slice1-5)) parts of the maxillary hard palate (Fig. 5A; Table 2, 3). Overall, the medially measured values (Hmv1-5) decreased from 0.72 ± 0.22 cm in the first measured slice (Hmv 1) to 0.66 ± 0.20 cm in the last slice (Hmv 5). The paramedially measured values decreased from the first to the last measured slice on the right and left side from 0.46 ± 0.21 cm and 0.45 ± 0.21 cm to 0.40 ± 0.20 cm and 0.40 ± 0.18 cm. Further differentiation of maxillary medial palatal thickness within the different skull configurations was determined. While no difference between the skeltal Class I, II or III could be observed, pronounced differences are present between the vertical phenotypes. A highly significant difference between the three vertical skull configurations regarding median palatal height was found in the first three measured layers (*p* < 0.001). This effect decreased slightly in the last two layers but was still significant (*p* < 0.01). An inverse effect could be observed in the paramedian layers. Initially, the differences concerning palatal thickness were significant (*p* < 0.01) in the first three layers and highly significant in the last two layers (*p* < 0.001) (Table 2, 3). The highest values were measured in patients with a brachyfacial phenotype (0.793 cm in the first to 0.729 cm in the last slice) as opposed to (0.685 cm in the first to 0.617 cm in the last slice) in dolichofacial and (0.674 cm in the first to 0.630 cm in the last slice) in mesofacial configurations. Those subjects with a brachyfacial skull configuration had the greatest median palatal thickness. Statistically significant differences between male and female patients were observed, while age did not significantly affect most measured slices (Table 2).

The median and paramedian thicknesses of the palatine bones (Hmd_15 und Hpm_lr_d_15) were pooled as described above, since no differences were observed between the different layers. A dependence on the vertical craniofacial type was observed for both parameters (*p* < 0.001), whereas skeletal Class I, II, and III relation had no influence whatsoever (Fig. 5).

Overall, patients with a brachyfacial facial skull configuration showed the highest palatal thickness (*p* < 0.001) in both the median (0.699 cm) and paramedian strata (0.327 cm). With regard to pairwise comparisons, patients with a brachyfacial configuration had a significantly greater median and paramedian thicknesses than mesofacial (median: 0.635 cm; paramedian: 0.253 cm) and dolichofacial (median: 0.643 cm; paramedian: 0.274 cm) patients (*p* < 0.001). Significant differences between mesofacial and dolichofacial were only present in the paramedian areas of the palatine bones (*p* = 0.003). Overall, sex-dependent differences could be observed for the median (*p* = 0.02) and paramedian (*p* = 0.003) parts of the palatine bone, with males yielding significantly higher values (Table 2). Age- dependent values were further observed, with the highest values measured for young patients between 20 and 40 years of age (Table 3).

**Table 2:** Demographic distribution with regard to sex of the measured hard and soft tissue parameters

	Female [*n* = 130]	Male [*n* = 110]	*p*	Total [*n* = 240]
Hmv1	0.662 +—0.176 (130)	0.782 +—0.241 (110)	0.022	0.717 +—0.217 (240)
Hmv2	0.638 +—0.168 (130)	0.765 +—0.237 (110)	0.004	0.696 +—0.212 (240)
Hmv3	0.629 +—0.164 (130)	0.743 +—0.229 (110)	0.005	0.681 +—0.205 (240)
Hmv4	0.609 +—0.159 (130)	0.715 +—0.215 (110)	0.010	0.658 +—0.194 (240)
Hmv5	0.609 +—0.186 (130)	0.717 +—0.220 (110)	< 0.001	0.658 +—0.209 (240)
Hpmlv1	0.392 +—0.161 (130)	0.527 +—0.222 (110)	< 0.001	0.454 +—0.202 (240)
Hpmlv2	0.383 +—0.157 (130)	0.507 +—0.213 (110)	< 0.001	0.440 +—0.195 (240)
Hpmlv3	0.373 +—0.153 (130)	0.491 +—0.209 (110)	< 0.001	0.427 +—0.190 (240)
Hpmlv4	0.359 +—0.148 (130)	0.477 +—0.202 (110)	< 0.001	0.413 +—0.184 (240)
Hpmlv5	0.350 +—0.150 (130)	0.460 +—0.202 (110)	< 0.001	0.400 +—0.184 (240)
Hpmrv1	0.393 +—0.165 (130)	0.533 +—0.232 (110)	< 0.001	0.457 +—0.210 (240)
Hpmrv2	0.379 +—0.160 (130)	0.515 +—0.230 (110)	< 0.001	0.441 +—0.206 (240)
Hpmrv3	0.365 +—0.158 (130)	0.498 +—0.221 (110)	< 0.001	0.426 +—0.201 (240)
Hpmrv4	0.358 +—0.159 (130)	0.483 +—0.217 (110)	< 0.001	0.415 +—0.197 (240)
Hpmrv5	0.347 +—0.156 (130)	0.468 +—0.217 (110)	< 0.001	0.402 +—0.196 (240)
Hmd_15	0.653 +—0.141 (650)	0.666 +—0.162 (550)	0.020	0.659 +—0.151 (1200)
Hpm_lr_d_15	0.276 +—0.120 (1300)	0.294 +—0.136 (1100)	0.003	0.284 +—0.128 (2400)
A_lr_15_v	1.062 +—0.125 (1300)	1.169 +—0.123 (1100)	< 0.001	1.111 +—0.135 (2400)
A_lr_15_d	1.286 +—0.152 (1300)	1.418 +—0.168 (1100)	< 0.001	1.347 +—0.172 (2400)
Ml_lr	4.312 +—0.463 (260)	4.673 +—0.442 (220)	< 0.001	4.478 +—0.487 (480)
Mt_lr	1.373 +—0.202 (260)	1.569 +—0.248 (220)	< 0.001	1.463 +—0.244 (480)
PL	5.230 +—0.346 (130)	5.632 +—0.353 (110)	< 0.001	5.414 +—0.402 (240)
S	3.134 +—0.301 (130)	3.368 +—0.343 (110)	< 0.001	3.241 +—0.341 (240)

Hmv1-5 = median thickness of the maxillary bone; Hpmlv1-5 = paramedian thickness the left maxillary bone PL = palatal plane; Hpmrv1-5 paramedian thickness the right maxillary bone; Hmd_15 = pooled median thickness of the palatine bone; Hpm_lr_d_15 =  = pooled paramedian thickness of the palatine bone; A_lr_15_v = pooled thickness of anterior alveolar crest; A_lr_15_d = pooled thickness of dorsal alveolar crest; Ml_lr = pooled length of the masseter; Mt_lr = pooled thickness of the masseter; PL = palatal length; S = length of the mid-palatal suture. All measurements are in cm.

**Table 3:** Demographic distribution with regard to age of the measured hard and soft tissue parameters

	Age [20–40) [*n* = 119]		Age [40–60) [*n* = 87]	Age [60–80) [*n* = 34]	*p*	Total [*n* = 240]
Hmv1	0.737 +—0.230 (119)		0.696 +—0.209 (87)	0.698 +—0.184 (34)	0.541	0.717 +—0.217 (240)
Hmv2	0.713 +—0.227 (119)		0.681 +—0.200 (87)	0.673 +—0.185 (34)	0.685	0.696 +—0.212 (240)
Hmv3	0.699 +—0.217 (119)		0.668 +—0.198 (87)	0.653 +—0.174 (34)	0.567	0.681 +—0.205 (240)
Hmv4	0.676 +—0.204 (119)		0.647 +—0.188 (87)	0.621 +—0.168 (34)	0.447	0.658 +—0.194 (240)
Hmv5	0.676 +—0.204 (119)		0.650 +—0.229 (87)	0.621 +—0.172 (34)	0.285	0.658 +—0.209 (240)
Hpmlv1	0.491 +—0.214 (119)		0.424 +—0.199 (87)	0.404 +—0.140 (34)	0.030	0.454 +—0.202 (240)
Hpmlv2	0.475 +—0.205 (119)		0.412 +—0.191 (87)	0.388 +—0.144 (34)	0.027	0.440 +—0.195 (240)
Hpmlv3	0.459 +—0.200 (119)		0.401 +—0.187 (87)	0.380 +—0.140 (34)	0.047	0.427 +—0.190 (240)
Hpmlv4	0.442 +—0.194 (119)		0.391 +—0.182 (87)	0.366 +—0.135 (34)	0.073	0.413 +—0.184 (240)
Hpmlv5	0.431 +—0.192 (119)		0.382 +—0.181 (87)	0.341 +—0.138 (34)	0.043	0.400 +—0.184 (240)
Hpmrv1	0.485 +—0.225 (119)		0.429 +—0.201 (87)	0.431 +—0.165 (34)	0.175	0.457 +—0.210 (240)
Hpmrv2	0.467 +—0.216 (119)		0.416 +—0.202 (87)	0.416 +—0.174 (34)	0.205	0.441 +—0.206 (240)
Hpmrv3	0.450 +—0.211 (119)		0.403 +—0.198 (87)	0.399 +—0.165 (34)	0.237	0.426 +—0.201 (240)
Hpmrv4	0.438 +—0.210 (119)		0.397 +—0.191 (87)	0.381 +—0.161 (34)	0.316	0.415 +—0.197 (240)
Hpmrv5	0.426 +—0.207 (119)		0.382 +—0.191 (87)	0.374 +—0.160 (34)	0.277	0.402 +—0.196 (240)
Hmd_15	0.663 +—0.164 (595)	0.651 +—0.139 (435)		0.664 +—0.134 (170)	0.618	0.659 +—0.151 (1200)
Hpm_lr_d_15	0.292 +—0.145 (1190)	0.283 +—0.107 (870)		0.260 +—0.106 (340)	0.011	0.284 +—0.128 (2400)
A_lr_15_v	1.140 +—0.117 (1190)	1.096 +—0.143 (870)		1.048 +—0.144 (340)	< 0.001	1.111 +—0.135 (2400)
A_lr_15_d	1.369 +—0.177 (1190)	1.330 +—0.167 (870)		1.310 +—0.158 (340)	< 0.001	1.347 +—0.172 (2400)
Ml_lr	4.534 +—0.471 (238)	4.414 +—0.492 (174)		4.445 +—0.515 (68)	0.035	4.478 +—0.487 (480)
Mt_lr	1.496 +—0.249 (238)	1.434 +—0.237 (174)		1.420 +—0.233 (68)	0.012	1.463 +—0.244 (480)
PL	5.447 +—0.414 (119)		5.364 +—0.395 (87)	5.430 +—0.378 (34)	0.152	5.414 +—0.402 (240)
S	3.263 +—0.347 (119)		3.219 +—0.346 (87)	3.224 +—0.312 (34)	0.718	3.241 +—0.341 (240)

Hmv1-5 = median thickness of the maxillary bone; Hpmlv1-5 = paramedian thickness the left maxillary bone PL = palatal plane; Hpmrv1-5 paramedian thickness the right maxillary bone; Hmd_15 = pooled median thickness of the palatine bone; Hpm_lr_d_15 =  = pooled paramedian thickness of the palatine bone; A_lr_15_v = pooled thickness of anterior alveolar crest; A_lr_15_d = pooled thickness of dorsal alveolar crest; Ml_lr = pooled length of the masseter; Mt_lr = pooled thickness of the masseter; PL = palatal length; S = length of the mid-palatal suture. All measurements are in cm. The pooled values ( Hpm_lr_d_15; A_lr_15_v; A_lr_15_) comprise 5 slices on the right and left side of the palate. The pooled values (Ml_lr and Mt_ lr) comprise one measurement on the right and left side of the jaw). All measurements are in cm.

The thickness of the alveolar ridge in the premolar region (A_lr_15_v) depended on both, skeletal Class I, II or III (*p* < 0.001) and vertical (*p* < 0.01) viscerocranial phenotype. Patients with a brachyfacial configuration had statistically significant higher values (mean thickness 1.124 cm) than mesofacial (*p* = 0.011) and dolicofacial (*p* = 0.012) phenotypes, both presenting with 1.105 cm. The thickness of the alveolar ridge in the molar region (A_lr_15_d) solely depends on the vertical viscerocranial phenotype (*p* < 0.001), with skeletal Class I, II or III having no influence. Patients with a brachyfacial relation have a significantly wider alveolar ridge (1.376 cm) than those with a dolicofacial (*p* < 0.001) (1.334 cm) or mesofacial (*p* < 0.001) (1.330 cm) configuration. However, no statistical difference was observed between dolicofacial and mesofacial configurations.

Alveolar ridge thickness is further associated with male sex and young age, with the highest values observed in male patients between 20 and 40 years of age (Table 2, 3).

The facial wall of the maxillary sinus and the pterygomandibular junction were not influenced by sagittal and vertical skull relations.

### Soft tissue parameters

**Fig. 6:**
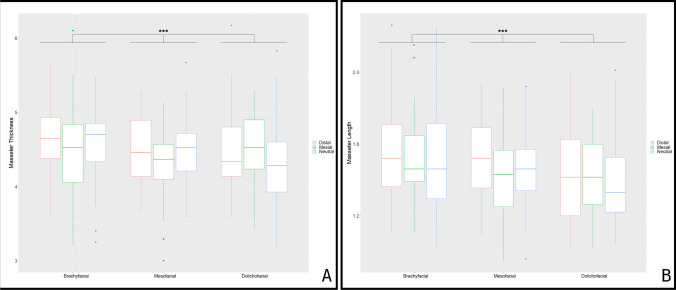
Masseter length and thickness in the different subgroups. The red-colored boxplot indicates distal, green mesial, and blue neutral skull configuration. ***p < 0.001

The masseter muscle’s length and thickness were equally pooled for the left and right sides of the face. Both, length and thickness seemed to be influenced by vertical viscerocranial phenotype (*p* < 0.001), with significantly longer and thicker muscles found in individuals with brachyfacial skull configuration (4.599 cm; 1.526 cm) than in those with dolicofacial (4.405 cm; 1.397 cm) phenotypes (*p* < 0.001) (Fig. 6). When comparing mesofacial (4.431 cm; 1.466 cm) and brachyfacial configurations, significant differences were only observed with regard to muscle length (*p* = 0.005), while significant differences between dolicofacial and mesofacial patients were observed with regard to muscle thickness (*p* = 0.027). Masseter length and thickness are also influenced by the respective skeletal Class (*p* < 0.05). Patients with skeletal Class II presented with a longer and thicker masseter (4.584 cm; 1.499 cm) than Class I (4.448 cm; 1.443) and Class III patients (4.398 cm; 1.456) (Fig. 6). Furthermore, sex had a highly significant influence on masseter length and thickness (*p* < 0.001) (Table 2). Also, an age-dependent influence could be observed, with the highest values for muscle length and thickness found in patients aged 20–40 (Table 3).

## Discussion

As bony and soft tissue landmarks are directly targeted during orthodontic treatment, a profound knowledge of midface anatomy is essential for orthodontists. In the maxilla, the T-zone has been established to safely place temporary anchorage devices (TADs), while the interradicular regions of both, the maxillary and mandibular buccal alveolar bones have proven to be equally suitable [21, 33, 34]. Since the correct placement is essential for the primary stability of TADs, the respective insertion regions are intensively studied in the literature. Overall, the literature reports on significant inter-individual differences with regard to palatal anatomy. These differences might be attributed to the fact that most studies do not record the respective viscerocranial phenotypes when evaluating the hard palate’s thickness. Therefore, we elaborated on the influence of vertical skull relations and the respective skeletal Class I, II or III configurations in great detail. While the skeletal Class I, II or III had no marked impact on palatal thickness, significant differences were observed between the three vertical subgroups, with the most significant palatal thickness found in brachyfacial skull configurations. On average, the median palatal thickness was 1 mm thicker in brachyfacial patients than in mesofacial or dolicofacial ones. Using surgical insertion guides for TADs has proven to increase placement accuracy, reducing early TAD loss. Those insertion guides might be essential in patients with mesofacial or dolicofacial skull configurations as opposed to brachyfacial patients, who present with more bone support in the anterior T- zone [35]. Palatal thickness significantly decreased from ventral to dorsal and from median regions of the palate to paramedian ones. These results agree with studies investigating TAD sites of the palatal posterior supra-alveolar bone [12, 17, 36]. In our collective of adult patients, age did not have a pronounced effect on the thickness of the hard palate. In contrast, statistically significant differences between male and female patients were observed (p < 0.01). This finding is congruent with sources stating gender-specific morphological variations, while other authors negate this [12, 17, 22, 34, 37].

Concerning maxillary body length, Class III patients proved to have a significantly shorter palate (p < 0.01) than Class I or Class II patients. With an average length of 5.313 cm, maxillary body length was reduced in Class III as opposed to Class I (5.406 cm) and Class II (5.404 cm) patients. Those results align with previous studies, which report a shorter maxillary body length in mesial configurations [1, 18, 38]. In our collective, the length of the mid-palatal suture is influenced by both, vertical skull configuration as well as skeletal Class I, II and III. Regarding vertical relations, brachyfacial patients had the longest suture, and dolicofacial patients presented with significantly shorter ones.

With reference to skeletal Class, and comparable to palatal length, the shortest mid-palatal sutures were found in Class III patients. A shorter mid-palatal suture in Class III patients might indicate less resistance to transversal expansion, compared to Class I and Class II patients. However, bone-anchored RME appliances in combination with maxillary protraction are beneficial in Class III patients for sagittal maxillary development [10, 39, 40]. The positive effect of bone-anchored RME in combination with maxillary protraction might be more critical in terms of stimulation of midface structures with distinct maxillary protraction protocols [39, 41].

Alveolar ridge thickness was also correlated with vertical skull relations, with brachyfacial patients presenting a significantly broader alveolar crest than those with a dolicofacial or mesofacial configuration, further underlining the influence of the viscerocranial phenotype on interindividual morphology. Hence, interradicular TADs might have a higher success rate in patients with brachyfacial skull configurations than those with dolicofacial or mesofacial ones. Concerning the measured soft tissue parameters, masseter thickness and length correlate with brachyfacial skull configurations, which goes along with the results of previously published data [26, 42].

## Conclusion

The findings of this study underline that besides sex and age, vertical skull configurations and skeletal Class I, II and III, as routinely obtained on lateral cephalometric images, influence soft and hard tissue landmarks of the skull. Clinical implications that could be derived from these findings might be within the scope of TAD placement. In the present study, patients with a dolicofacial skull configuration and skeletal Class III present with a significantly shorter maxilla and mid-palatal suture, as well as reduced palatal height and alveolar ridge thickness, combined with masticatory muscles, which are reduced in length and thickness. These factors could necessitate shorter implants and lower orthodontic forces to achieve clinical treatment success. Furthermore, in knowledge of the respective morphology, conventional and cortically anchored orthodontic appliances could be tailored to individual needs.

## Limitations

Nevertheless, this study also presents some limitations primarily associated with the retrospective study design. While selecting the patients from the local Department of Radiology archives leads to a large sample size and sufficient power of the presented measurements, no records of an initial orthodontic treatment could be obtained. The distribution of the patient collective into the cephalometric subgroups was nonetheless conducted and should therefore be interpreted as a proof of principle design. Further prospective studies with orthodontic pre-treatment records are needed to validate these findings.
